# Review Analysis of the Association between the Prevalence of Activated Brown Adipose Tissue and Outdoor Temperature

**DOI:** 10.1100/2012/793039

**Published:** 2012-04-19

**Authors:** Yung-Cheng Huang, Chien-Chin Hsu, Pei-Wen Wang, Yen-Hsiang Chang, Tai-Been Chen, Bi-Fang Lee, Nan-Tsing Chiu

**Affiliations:** ^1^Department of Nuclear Medicine, Kaohsiung Chang Gung Memorial Hospital and Chang Gung University College of Medicine, Kaohsiung 83301, Taiwan; ^2^Department of Information Engineering, I-Shou University, Kaohsiung, Taiwan; ^3^Department of Medical Imaging and Radiological Sciences, I-Shou University, Kaohsiung 82445, Taiwan; ^4^Department of Nuclear Medicine, National Cheng Kung University Hospital, College of Medicine, National Cheng Kung University, Tainan 70428, Taiwan

## Abstract

Brown adipose tissue (BAT) is important for regulating body weight. Environmental temperature influences BAT activation. Activated BAT is identifiable using ^18^F-fluorodeoxyglucose positron emission tomography/computed tomography (^18^F-FDG PET/CT). ^18^F-FDG PET/CT scans done between June 2005 and May 2009 in our institution in tropical southern Taiwan and BAT studies from PubMed (2002–2011) were reviewed, and the average outdoor temperatures during the study periods were obtained. A simple linear regression was used to analyze the association between the prevalence of activated BAT (*P*) and the average outdoor temperature (*T*). The review analysis for 9 BAT studies (*n* = 16, 765) showed a significant negative correlation (*r* = −0.741, *P* = 0.022) between the prevalence of activated BAT and the average outdoor temperature. The equation of the regression line is *P*(%) = 6.99 − 0.20 × *T*  (°C). The prevalence of activated BAT decreased by 1% for each 5°C increase in average outdoor temperature. In a neutral ambient temperature, the prevalence of activated BAT is low and especially rare in the tropics. There is a significant linear negative correlation between the prevalence of activated BAT and the average outdoor temperature.

## 1. Introduction

Brown adipose tissue (BAT), with its thermogenic potential contributing to energy expenditure, is believed to influence body weight and age-related metabolic diseases [1, 2]. It is potentially a candidate target tissue for anti-obesity therapies and has recently attracted much attention. BAT is abundant in newborns and helps protect them from lethal hypothermia [3]. In spite of the decrease in the amount of BAT with age, islets of brown adipocytes still endure in the white adipose tissue of adult humans [3, 4]. The presence of this BAT, the recruitment of BAT, and the conversion of white into brown adipocytes may contribute to the development of new treatments for the current obesity pandemic [5, 6]. Temperature-dependent BAT activation might be of interest in future approaches against obesity.

The primary thermoregulatory stimulus for activating BAT is a reduction in skin or external temperature [7]. BAT activation is more frequent during the cooler seasons of the year [8–10] and can be detected using ^18^F-fluorodeoxyglucose (^18^F-FDG) positron emission tomography (PET). Previous studies of the occurrence of activated BAT detected using ^18^F-FDG PET are limited to the temperate zone, for example, North America and Europe. To further recognize its occurrence in tropical areas and to investigate the relationship between the prevalence of activated BAT and outdoor temperature over a wide range, we did a review analysis of data from our own patients and relevant studies from the PubMed database.

## 2. Materials and Methods

### 2.1. Participants

We reviewed ^18^F-FDG PET/CT scans done between June 2005 and May 2009 in our institution in tropical southern Taiwan (located at 22.7°N). The patients fasted for at least 6 hours and were then intravenously injected with ^18^F-FDG. No attempt was made to prevent BAT activation before the PET/CT scan by removing the cold stimulus from controlling the patient's environmental temperature or prescribing any medication such as beta blockers or diazepam.

Each patient was intravenously injected with 370–555 MBq (10–15 mCi) of ^18^F-FDG and the dose was adjusted according to body weight in pediatric patients. Thereafter they stayed calmly in the supine position for 1 hour in an isolated, continuously air-conditioned room. An integrated PET/CT scanner (Discovery ST, GE Healthcare) was used to acquire images from the head to the upper part of the thighs. The images were reconstructed with an ordered-subset expectation maximization algorithm (OSEM, 2 iterations, 30 subsets). The transaxial PET data were obtained as 128 × 128-pixel images with a slice thickness of 3.27 mm. Coronal and sagittal sections, as well as maximum intensity projection PET images, were also reformatted for PET/CT imaging fusion and interpretation.

**Figure 1:**
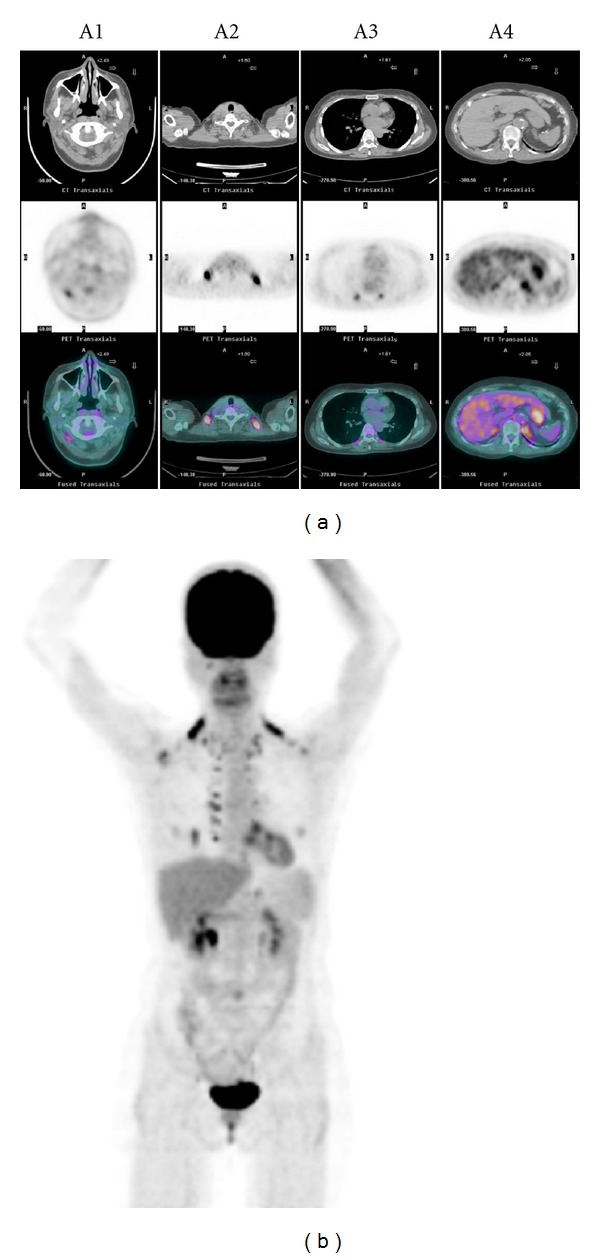
The ^18^F-FDG PET/CT scan of a patient with activated BAT displayed on transverse slice of CT (a: upper row), PET (a: middle row), a fusion of PET and CT images (a: lower row), and a maximum intensity projection (b); hypermetabolic BAT deposits of increased ^18^F-FDG uptake with a symmetric distribution in the bilateral posterior neck (A1), supraclavicular (A2), paravertebral (A3), and suprarenal (A4) areas.

^18^F-FDG PET/CT scans with reports stating that the patients had activated BAT were reviewed by 2 experienced nuclear medicine physicians to confirm the presence of activated BAT. Activated BAT was considered present if there were areas of increased ^18^F-FDG uptake corresponding to the CT density of adipose tissue (−250 to −50 Hounsfield units) and compatible with characteristic patterns of BAT distribution (Figure 1). This retrospective study was approved by our hospital's Institutional Review Board with a waiver of consent.

### 2.2. Literature Search

We searched the PubMed database using the medical subject headings *adipose tissue, brown*, and *(fluorodeoxyglucose F18 or positron-emission tomography)* and retrieved English-language articles published from January 2002 through June 2011. We looked for studies that detected BAT using ^18^F-FDG PET. Criteria for exclusion included nonhuman data; case reports; not a full paper; specialized subjects (such as pediatric patients, or only men or women); patients with a specific disease, with premedication or controlled temperature to influence BAT activation, repetitive data, and patients with BAT in a restricted anatomic distribution. Studies had to include definite information on the prevalence of activated BAT, where the study was done, and the months during which it was done.

### 2.3. Temperature Data and Statistical Analysis

For our patients, we obtained the average outdoor temperature data from the Taiwan Central Weather Bureau. After we had identified the most relevant studies, we obtained the cities' average monthly temperatures during the respective study periods of interest from the official weather department websites of the countries in which the studies had been done. The average outdoor temperatures during the respective study periods were therefore acquired. We also grouped the ^18^F-FDG PET/CT scans from our patients with activated BAT according to the season in which the PET/CT was done.

A simple linear regression was then done to analyze the association between the prevalence of activated BAT (*P*) and the average outdoor temperature (*T*) during the respective study periods. SPSS 17 for Windows (SPSS Inc., Chicago, IL, USA) was used for the statistical analysis. Significance was set at *P* < 0.05.

## 3. Results

**Figure 2:**
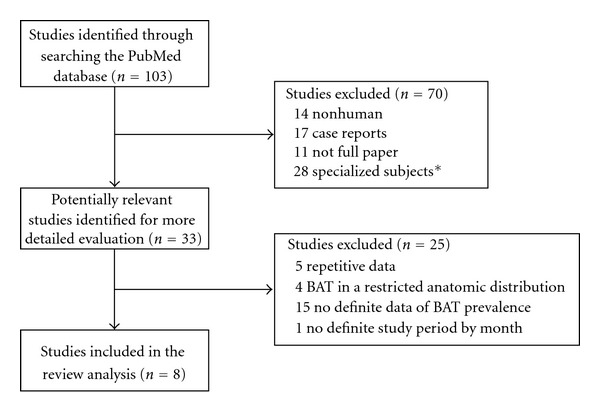
The flow chart for the inclusion and exclusion of studies for the current review analysis (*8 on pediatric patients, 2 on only men or women, 8 on patients with a specific disease, 10 on premedication or temperature control to influence BAT activation).

**Figure 3:**
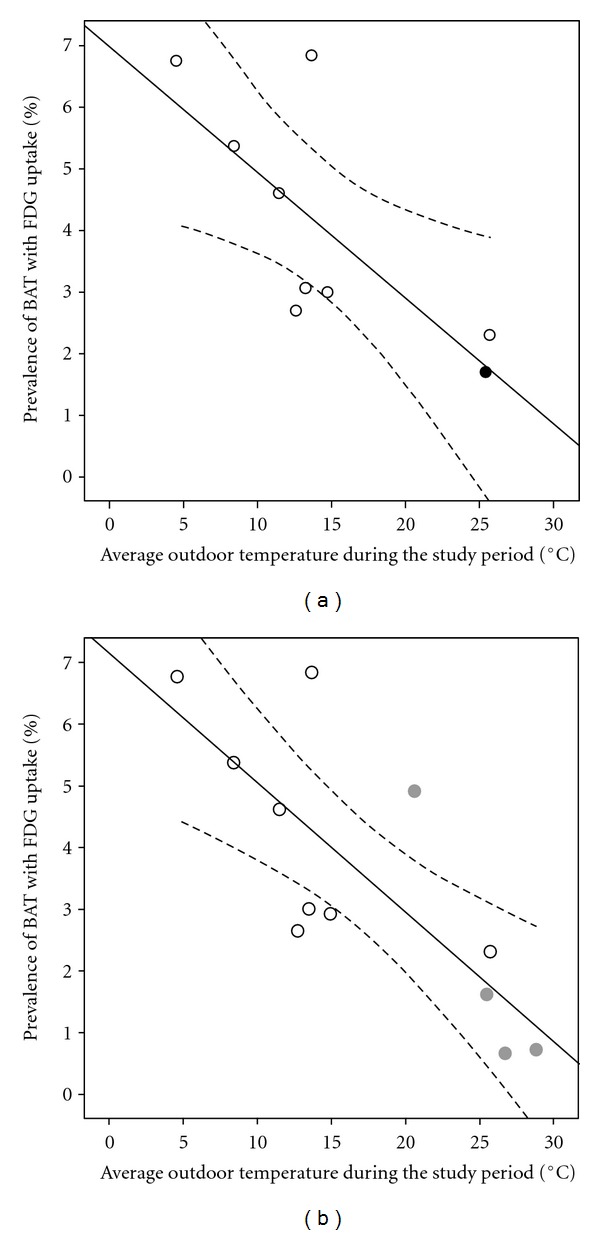
A simple linear regression was done for the review analysis of the association between the prevalence of activated BAT with ^18^F-FDG uptake and the average outdoor temperatures during the study period. (a) The prevalence of brown adipose tissue (BAT) in the 8 previous studies [8–15] (white circles) and our patients (black circle), plotted against the average outdoor temperatures during the study period. There was a significant negative correlation (*r* = −0.741, slope = −0.20, *P* = 0.022) between the prevalence of activated BAT on ^18^F-FDG PET scans and the average outdoor temperature. (b) The prevalence of BAT in the 8 previous studies (white circles) and our data grouped into 4 seasons (gray circles) plotted against the average outdoor temperature during the study period. The correlation was more significant (*r* = −0.792, slope = −0.21, *P* = 0.002). The solid line is the linear regression line; dashed lines indicate the 95% confidence intervals.

In our hospital, 1740 patients underwent 1903 consecutive clinical ^18^F-FDG PET/CT scans for a variety of purposes between June 2005 and May 2009. Activated BAT was identified on 37 scans (1.94%, 37/1903) from 30 patients (1.73%, 30/1740; male: 0.39%, 4/1017; female: 3.60%, 26/723; mean age: 40.6 years; range: 12–73 years). The PubMed search yielded 103 studies evaluating BAT with ^18^F-FDG PET. Seventy of these studies met at least 1 of the exclusion criteria and were rejected. Of the remaining 33 studies, 25 were considered ineligible after the full article was reviewed (Figure 2). Eight studies fulfilled all of the inclusion criteria (Table 1) [8–15]. Seven had been done in North America and Europe, and 1 in Turkey. For the 9 cohorts, including our patients, and the 8 relevant published reports (study *n* range: 638–4842 patients; total: 16,765 patients analyzed), there was a significant negative correlation (*r* = −0.741, slope = −0.20, *P* = 0.022) between the prevalence of activated BAT on ^18^F-FDG PET scans and the average outdoor temperature (Figure 3(a)). The equation of the regression line is *P*  (%) = 6.99 − 0.20 × *T* (°C). Subgrouping the 37 PET/CT scans from our patients with activated BAT according to the season in which the PET/CT was done supporting this correlation (Figure 3(b); *r* = −0.792, slope = −0.21, *P* = 0.002). Based on this regression line, the prevalence of activated BAT decreased by 1% for every 5°C increase in average outdoor temperature.

## 4. Discussion

We found that the prevalence of ^18^F-FDG PET-detected activated BAT was very low and varied considerably from 1.72% (for our patients living in a tropical climate) to 6.85% (for the other 8 cohorts in the reviewed literature) [8–15]. In the review analysis over a wide range of outdoor temperature, a simple linear regression analysis of all 9 cohorts showed a significant negative correlation between the prevalence of activated BAT and the average outdoor temperature during the study period. For each 5°C increase in average outdoor temperature, the prevalence of ^18^F-FDG PET-detected activated BAT decreased by 1%. This reinforces the importance of outdoor temperature for activating BAT and provides an estimation of the prevalence of activated BAT based on the outdoor temperature. In a thermoneutral environment, the formula from the result of this review analysis offers a baseline for reference and comparison.

Previous studies have shown the influence of environmental temperature on the activation of BAT. By controlling the environmental temperature in a study with 56 healthy volunteers [16], the prevalence of activated BAT increased to 33% after the participants had been exposed to cold temperatures in the form of an intermittently applied ice-cooled footrest and a cool environmental temperature of 19°C. In contrast, in a study on children [17], the prevalence of activated BAT decreased by two-thirds when the environmental temperature rose from 21°C to 24°C. In the current review, the two cohorts with the lowest prevalence of activated BAT were ours and the one in Yeung et al. [12]. The ^18^F-FDG PET scans in the latter were done in New York City in July and August 2002, the hottest period in the temperate zone, when the temperature is similar to the high annual average temperature in tropical areas. We also found a seasonal variation in the prevalence of activated BAT that was consistent with other studies [8, 9] and mainly due to the effect of the seasonal outdoor temperature. Further subgrouping the PET/CT scans from our patients with activated BAT according to the four seasons resulted in a more significant negative correlation and strengthened the regression relationship between activated BAT prevalence and outdoor temperature.

A recent study [18] with histological analysis found a high prevalence of BAT in adult humans, and the activated BAT detected by ^18^F-FDG PET displayed strong immunoreactivity for uncoupling protein 1 (UCP1). UCP1 uncouples adenosine-5′-triphosphate (ATP) synthesis from substrate oxidation in BAT to dissipate the electrochemical gradient as heat and is necessary for norepinephrine-induced glucose utilization [19]. Its activity depends on the availability of fatty acids delivered upon BAT's beta-adrenergic activation, which, physiologically, ensues from the sympathetic nervous system activation of the tissue [20]; exposure to cold is one of the most influential factors. Exposure to cold causes sympathetic stimulation of BAT, after which the cold stimulated BAT perfusion dependently dissipates energy, increases glucose utilization, and increases glucose transporter (GLUT) expression [21, 22]. An increased affinity for or activation of the GLUT1 isoform is responsible for the norepinephrine-induced increase in glucose transport in brown adipocytes, and that is likely mediated by intracellular cAMP [23]. GLUT4 and UCP1 are more highly expressed in BAT than in white adipose tissue. Exposure to cold also increases the mRNA levels of GLUT4, an isoform of glucose transporters expressed in insulin-sensitive tissues in BAT [24]. These cellular components and molecular mechanisms may contribute to elevated ^18^F-FDG accumulation in cold-stimulated BAT.

The limitations of this study must be addressed. The review study is retrospective in nature. The average room temperatures (indoor) at which patients did their daily lives and received PET examinations as well as for how long they had been exposed to that particular temperature were not available for analysis. The average outdoor temperature during the study period we obtained cannot represent the actual temperature around every patient before their PET scan. However, we checked the actual daily temperature on every scan performed of our patients and the average was 25.3°C, which was very close to the average monthly temperature of 25.4°C during our study period. The difference is acceptable. Because of the diverseness of the published studies, we focused only on the outdoor temperature, the most important factor for BAT activation. Other factors that can influence BAT activation were not appropriately evaluated by this review analysis; however, our results provide an estimation of the occurrence of activated BAT for clinical ^18^F-FDG PET practice.

## 5. Conclusion

In a neutral ambient temperature, the prevalence of activated BAT is low and especially rare in the tropical areas. In this review analysis, we found a significant negative correlation between the prevalence of activated BAT and the average outdoor temperature during the study period.

##  Conflict of Interests

The authors have no conflict of interests to declare.

## Figures and Tables

**Table 1:** Prevalence of activated BAT (detected by ^18^F-FDG uptake) and average outdoor temperature during the study period.

Data source	Prevalence of BAT (%)	Study period	Average outdoor temperature (°C)
Hany et al., Zurich, Switzerland [11]	2.66 (17/638)	04/2001–11/2001	12.7
Cohade et al., Baltimore, USA [8]	6.85 (62/905)	07/2001–06/2002	13.7
Yeung et al., New York, USA [12]	2.32 (20/863)	07/2002–08/2002	25.7
Kim et al., New York, USA [13]	3.02 (35/1159)	03/2000–11/2003	13.4
Cypess et al., Boston, USA [14]	5.38 (106/1972)	08/2003–05/2006	8.4
Au-Yong et al., Nottingham, UK [9]	4.62 (167/3614)	03/2006–10/2008	11.5
Ouellet et al., Québec, Canada [10]	6.77 (328/4842)	01/2007–12/2008	4.6
Akkas et al., Ankara, Turkey [15]	3.00 (31/1032)	01/2008–10/2008	14.7*
Our data, Kaohsiung, Taiwan	1.72 (30/1740)	06/2005–05/2009	25.4
Winter	4.92 (21/427)		20.6
Spring	1.62 (10/618)		25.5
Summer	0.73 (3/413)		28.8
Autumn	0.67 (3/445)		26.7

The average outdoor temperatures during the study periods were obtained from the Federal Office of Meteorology and Climatology MeteoSwiss, the USA National Oceanic and Atmospheric Administration, Met Office Hadley Centre Central England Temperature Data, the National Climate Data and Information Archive of Canada, Turkish State Meteorological Service, and the Central Weather Bureau of Taiwan (*coordinated with http://www.geodata.us).
